# Roller Bearing Fault Diagnosis Based on Nonlinear Redundant Lifting Wavelet Packet Analysis

**DOI:** 10.3390/s110100260

**Published:** 2010-12-28

**Authors:** Lixin Gao, Zijing Yang, Ligang Cai, Huaqing Wang, Peng Chen

**Affiliations:** 1 Key Laboratory of Advanced Manufacturing Technology, Beijing University of Technology, Chao Yang District, Beijing, 100124, China; E-Mail: lead0003@163.com (G.L.); 2 School of Mechanical & Electrical Engineering, Beijing University of Chemical Technology, Chao Yang District, Beijing, 100029, China; 3 Graduate School of Bioresources, Mie University/1577 Kurimamachiya-cho, Tsu, Mie, 514-8507, Japan; E-Mail: chen@bio.mie-u.ac.jp

**Keywords:** roller bearings, nonlinear, redundant lifting wavelet packet, fault diagnosis

## Abstract

A nonlinear redundant lifting wavelet packet algorithm was put forward in this study. For the node signals to be decomposed in different layers, predicting operators and updating operators with different orders of vanishing moments were chosen to take norm *l^p^* of the scale coefficient and wavelet coefficient acquired from decomposition, the predicting operator and updating operator corresponding to the minimal norm value were used as the optimal operators to match the information characteristics of a node. With the problems of frequency alias and band interlacing in the analysis of redundant lifting wavelet packet being investigated, an improved algorithm for decomposition and node single-branch reconstruction was put forward. The normalized energy of the bottommost decomposition node coefficient was calculated, and the node signals with the maximal energy were extracted for demodulation. The roller bearing faults were detected successfully with the improved analysis on nonlinear redundant lifting wavelet packet being applied to the fault diagnosis of the roller bearings of the finishing mills in a plant. This application proved the validity and practicality of this method.

## 1. Introduction

With the continuous changes and improvements of modern science, technologies and industries, various kinds of mechanical equipments are developing rapidly towards the trend of large scale, high precision, high speed and automation. With the increasingly meticulous design and manufacturing of equipment, the social and economic benefits created by them have also been accumulating. However, the normal equipment operation inevitably causes the dissipation of components, the long-term accumulation of which will eventually cause the failure of the whole equipment. Due to the difference in the production and assembly of equipments as well as the complexity of the operation environment, there are generally many uncertainties during the operation. In order to prevent the occurrence of accidental faults and avoid the resulting severe consequences, the investigation and application of advanced signal processing techniques and the achievement of the effective monitoring of equipment status is of great practical significance.

Equipment in operation generally exhibits nonlinear engineering characteristics, so the wavelet transform has been widely applied to the fault diagnosis of equipment owing to its multi-resolution analysis feature. However, conventional wavelet functions are generally constructed in the field of mathematics and have difficulties in fitting with practical engineering signals; besides, wavelet functions in different scales are acquired from mother wavelets after scaling and translation. Therefore, once a wavelet function is chosen, identical filter groups are employed both within one scale and among different scales, which suggests a lack of flexibility and certain limitation in capturing variable information.

In 1996, Sweldens from Bell Laboratories proposed a lifting framework to construct compactly supported wavelets and dual wavelet functions in the time domain. Wavelet functions with expected characteristics could be obtained with the design on the lifting operator based on prior initial biorthogonal filter groups, e.g., increasing the orders of vanishing moments of wavelets or making wavelet functions to approximate specific waveforms [1]. Such a wavelet construction method, which does not depend on the Fourier transform but is implemented completely in the time domain, is also known as the second generation of wavelet transforms. With its advantage of simple and rapid calculation, not only the characteristics of first-generation wavelets could be preserved, but the drawbacks of the scaling and translation invariation could be overcome [2]. The lifting algorithm has been widely studied since the day of its presentation. Claypoole *et al.* presented a design method of the predicting operator and updating operator based on equivalent filters [3]; Duan *et al.* applied the sliding-window characteristics extraction method based on the lifting algorithm and successfully detected the shock components induced by misalignment, imbalance and fracture of bearing pads [4]; Huang *et al.* extracted distribution information and composed the feature vectors by applying sampling-importance-resampling procedure to the signals decomposed with lifting wavelet packets in the wavelet domain, then the equipment state was evaluated with the support vector machine [5]. Samuel *et al.* suggested an adaptive lifting algorithm with constraints to detect and diagnose the faults of the bearings in an epicyclic gearbox of helicopter transfer equipment [6]. Because lifting wavelets had the translation invariability, Lee *et al.* put forward the non-sampling lifting wavelet transform and omitted the link of subdivision in original transform [7]. To explain the propagation of error in the redundant lifting algorithm, Li *et al.* presented an improved redundant lifting algorithm based on normalized factors, and extracted successfully the characteristics of faint fault signals using the shock pulse method [8]. Zhou *et al.* applied the second-generation redundant wavelet transform in the vibration signal analysis of the gearboxes and the valve gears of petrol engines, and conducted identification using extracted fault characteristics as the classifier input, with better classification results being obtained [9]. In conclusion, the lifting algorithm has been constantly investigated in depth and applied to signal analysis, image processing and other fields. Based on prior studies, the thought of nonlinear redundant lifting wavelet packet transform was introduced for the first time in this paper. Meanwhile, combined with solutions of frequency alias and band interlacing problems, a new nonlinear redundant lifting wavelet packet transform decomposition and node-signal single-branch algorithm was then proposed and successfully applied to the fault diagnosis of the roller bearings in large equipment.

The paper is organized as follows: Section 2 presents the implementation of the nonlinear redundant lifting algorithm in detail, with the problems of frequency alias and band interlacing in the algorithm being analyzed and improved; Section 3 presents a characteristics extraction method based on the wavelet packet energy and single-branch reconstruction signal demodulation; in Section 4, the algorithm is validated through the analysis of engineering examples; finally, conclusions are made.

## 2. Improved Algorithm for Nonlinear Redundant Lifting Wavelet Packet

### 2.1. Lifting Wavelet Principle

The lifting wavelet transform is implemented in the following three steps [10]:

1. *Split*. The original signal *X* = {*x*(*k*), *k* ∈ *Z*} is subdivided into two parts: odd sample *x_o_* (*k*) and even sample *x_e_* (*k*):
  (1) $$x_{e}(k)=\{x(2k),k\in Z\},\;\;\;x_{o}(k)=\{x(2k+1),k\in Z\}$$
2. *Prediction*. Since adjacent signal samples are highly correlated, the odd sample is predicted based on the even sample through a predicting operator *P*, and the prediction error is defined as the detail signal *d(k)*:
  (2) $$d(k)=x_{o}(k)-P[x_{e}(k)]$$
3. *Updating*. In order to reduce the frequency alias induced by the down-sampling in the splitting process and correct the difference between *x_e_(k)* and *X*, it is necessary to update the detail signal *d(k)* through an updating operator *U* and replace *x_e_(k)* so as to acquire a smoother approximation signal *d(k)*:
  (3) $$a(k)=x_{e}(k)+U[d(k)]$$

Since the lifting wavelet transform is performed completely in the time domain, the reconstruction course is very simple, including updating recovery, prediction recovery and merging, *i.e.*, the direction of the signal flow and the operator in the original formula are reversed.

### 2.2. Redundant Lifting Wavelet Principle

Although the lifting algorithm has been widely used, it still has the following problems:

1. The first step of the lifting wavelet transform is to perform a subdivision which is actually a down-sampling course, so the lengths of the acquired odd and even samples are both the half of original signals. With the increase of the decomposition scale, the point number of samples decreases constantly, and the amount of the information provided decreases consequently.
2. Since the split is a down-sampling course, the sampling rate of detail signals may no longer satisfy the Nyquist sampling principle. Accordingly, frequency alias emerges, and false frequency components are created.
3. Due to the existence of the split course, the output results change when original signals delay for an odd number of sampling points. Therefore, the lifting algorithm does not have the translation invariability.

**Figure 1.**
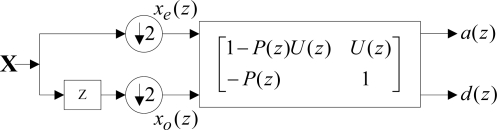
Expression of multi-phase matrix for lifting wavelets.

According to the analysis, all the above problems are induced in the link of split. Accordingly, the split step was considered to be removed. The representation of multi-phase matrix for the lifting wavelet transform was shown in Figure 1 [11]:

With the equivalent translocation transform [12] being performed on the above figure and the down-sampling step being removed, translation was performed on detail signals, then the decomposition course of the redundant lifting wavelet transform divided into the following two steps [13]:

1. *Prediction*:
  (4) $$d(k)=x(k)-P_{\mathrm{new}}[x(k)]$$
2. *Updating*:
  (5) $$a(k)=x(k)+U_{\mathrm{new}}[d(k)]$$

The reconstruction course of the redundant lifting algorithm still included three steps: updating recovery, prediction recovery and merging. The approach to the implementation of updating recovery and prediction recovery was the same as that to the lifting algorithm, but the course of acquiring reconstructed signals by merging was changed into the process of averaging the samples *x^u^(k)* and *x^p^(k)* acquired by updating recovery and prediction recovery, *i.e.*:

(6) $$x(k)=\frac{1}{2}\cdot [x^{u}(k)+x^{p}(k)]$$

According to (4) and (5), the algorithm *á trous* [12] was introduced, and a method of designing the predicting operator and the updating operator of redundant lifting wavelets could be obtained; the initial prediction and updating coefficients could be acquired with the interpolating subdivision method; the coefficient of layer *j* was obtained through the interpolation zero-filling method performed on the coefficient of layer *j* − *1*. Therefore, the predicting operators and updating operators used for decomposition at different layers were all different; besides, the lengths of the approximation signal samples and detail signal samples acquired from decomposition were the same as those of original signals, so the information was redundant.

### 2.3. Nonlinear Redundant Lifting Algorithm

The construction of wavelet functions based on the lifting algorithm is conducted completely in the time domain rather than on a basic function after scaling and translation, which made it possible to design different predicting operators and updating operators for one same decomposition layer or different decomposition layers. Claypoole *et al.* put forward a nonlinear lifting algorithm based exactly on the above idea, *i.e.*, selecting different predicting operators according to the local characteristics of images [14]. In the local smooth area of an image, the adjacent samples had strong correlation, so predicting operators with high-order vanishing moments were employed; near the edge of an image, the adjacent samples had weak correlation, so low-order predicting operators were employed. Accordingly, such lifting wavelet transform, in which predicting operators were determined based on the sample correlation, was nonlinear, while the lifting algorithm guaranteed the reversibility of the transform.

Based on the above idea and prior studies, the idea of a nonlinear transform was introduced in the redundant lifting wavelet packet transform in this study to obtain a nonlinear redundant lifting wavelet packet algorithm. Since the nodes generated by the decomposition of wavelet packets involved different band information, predicting operators and updating operators with different orders of vanishing moments were employed when performing redundant lifting wavelet packet decomposition on the node signals to be decomposed in layer *j (j* ≥ *1)*, so that all the characteristic information in the signals to be decomposed could be matched as much as possible. Because the number of node signals in the wavelet packet of layer *j*, 2*^j^* times of selection of predicting operators and updating operators were needed.

#### 2.3.1. Design of Predicting Operators and updating Operators

In this study, the initial prediction coefficients and updating coefficients in all layers were designed with interpolating subdivision before *á trous* was introduced to perform zero-filling interpolation on initial coefficients. For the decomposition at layer *j*, 2*^j^* − *1* zeros were interpolated among initial prediction coefficients and updating coefficients to acquire the prediction coefficients and updating coefficients at layer *j*.

After their respective design, the predicting operators and updating operators (for decomposition) with different lengths were chosen according to the time-frequency characteristics of the scale function and wavelet function. The length of the predicting operator was denoted by *N*, and the length of the updating operator was denoted by *Ñ*. When *N* was small, the frequency characteristics of the scale function and wavelet function could not be improved even if *Ñ* was increased; when *N* increased gradually, the frequency characteristics of the scale function and wavelet function would be improved [13]; besides, when *N* increased inapparently, the frequency characteristics of the scale function and wavelet function did not improve much. Because of the above two reasons as well as the purpose of reducing the amount of computation, *N* = 4, 12, 20 and *Ñ* = 4, 12, 20 (*Ñ* ≤ *N*) were chosen as the lengths of predicting operators and updating operators in this study, respectively. Thus, the following six wavelet functions in total could be obtained through combination:

**Table 1.** Selection of predicting operators and updating operators.

**Predicting operators**	4	12	12	20	20	20
**Updating operators**	4	4	12	4	12	20

Therefore, six groups of decomposition results could be obtained from the node signals of each wavelet packet for each decomposition result. The answer as to which pair of predicting operator and updating operator generated by corresponding decomposition result was optimal depended on the established objective function.

#### 2.3.2. Norm *l^p^*

After being decomposed with the redundant lifting wavelet packet algorithm, the signals could be characterized by a series of approximation coefficients and wavelet coefficients. In the various application fields of wavelets, such as fault signal analysis, signal denoising and image compression, it is generally preferred that the number of non-zero wavelet coefficients is as small as possible. Because the wavelet transform is flexible in basis selection while the time-domain structure characteristics of wavelets based on the lifting algorithm bring more freedom in selecting predicting operators and updating operators, which wavelet basis is the optimal one matching the characteristics of signals and satisfying analysis requirements? Since the wavelet transform is the inner product operation between signals and wavelet function and the autocorrelation function and cross-correlation function of the signals can be expressed by inner product form, the wavelet transform could be regarded as a measure for the correlation or similarity between the wavelet function and signals [15]. The more similar the selected wavelet function is to the interested characteristics in signals, the larger the wavelet coefficient will be; consequently, such characteristics could be embodied more significantly and other components in signals were inhibited. Therefore, the maximal similarity to signal characteristics could be used as the criterion for selecting the optimal wavelet basis. However, the following problem emerges: how to measure the similarity between signals and the wavelet basis? Since the purpose of the wavelet transform is to characterize original signals with a few wavelet coefficients, the sparsity could be used as one of the criteria for the similarity assessment [16].

There are multiple parameters used for the sparsity evaluation. For the case without noise, generally norm *l^0^* (*i.e.*, the number of non-zero elements in the data vector) or the *Shannon* entropy standard is used to measure the sparsity of samples; for the case with noise, other parameters should be selected because the introduction of weaker noise is more likely to turn original sparse samples into ones that are not sparse at all [17]. A frequently used approach is to replace norm *l^0^* with *l^p^*. *l^p^* is defined as follows:

(7) $${\left\|x\right\|}_{p}={\left(\sum_{k}{\left|x_{k}\right|}^{p}\right)}^{1/p},\;\;\;\;p\leq 1$$

Norm *l^0^* is the utmost value of norm *l^p^* when *p→0*. In order to enable *l^p^* to approach *l^0^* as much as possible, generally *p* is set very low, so *p* is chosen as 0.1 in this study. Besides, predicting operators and updating operators with different lengths were used in this study to perform redundant lifting wavelet packet decomposition on signals. The more similar the interesting components in signals were to the wavelet function corresponding to one of the groups of predicting operators and updating operators, the larger wavelet coefficients were acquired. According to the law of conservation of energy, the wavelet coefficients for other components in signals would be smaller or even approach zero. Thus, the number of the non-zero elements in wavelet coefficients would decrease, the coefficients became sparser, and corresponding norm *l^p^* would be lower. Therefore, the predicting operator and updating operator corresponding to the minimal *l^p^* of the coefficients acquired through decomposition were the optimal operators in this study.

For computation simplification and the convenience of comparison, the coefficients acquired through the decomposition of wavelet packets were normalized to solve *l^p^*. Suppose the node signals in the wavelet packets to be decomposed at layer *j−1* were *x_j−1,m_* *(m = 1,2…2* *^j−1^)*, then the wavelet packet coefficient for layer *j* was *x_j,n_* *(n = 1,2…2* *^j^)*. Normalized *l^p^* was solved against *x_j,n_*, *i.e.*:

(8) $${\left\|x_{j,n}\right\|}_{p}={\left(\sum_{k}{\left|x_{j,n,k}/\sum_{k}x_{j,n,k}\right|}^{p}\right)}^{1/p},\;\;\;\;p\leq 1\;;\;n=1,2,\cdots 2^{j}$$

where *x_j,n,k_* was the No. *k* element in wavelet packet coefficient No. *n* at layer *j*. Since low-frequency and high-frequency decomposition was conducted simultaneously in wavelet packet decomposition:

(9) $${\left\|x_{j-1,m}\right\|}_{p}={\left\|x_{j,2m-1}\right\|}_{p}+{\left\|x_{j,2m}\right\|}_{p},\;\;\;\;p\leq 1\;;\;m=1,2,\cdots 2^{j-1}$$

where ||*x*_*j*−1,*m*_||*_p_* was the normalized *l^p^* of the node signals in No. *m* wavelet packet at decomposed layer *j* − 1. Because six groups of wavelet functions were chosen to decompose *x*_*j*−1,*m*_ in this study, six ||*x*_*j*−1,*m*_||*_p_* could be obtained for each *x*_*j*−1,*m*_. The group of predicting operators and updating operators corresponding to the minimal value was selected as the optimal operators.

In conclusion, the nonlinear redundant lifting wavelet packet algorithm could be divided into the following five steps:

1. The number *i* of decomposed layers was determined;
2. Totally six groups of wavelet functions with different vanishing moments were chosen to perform wavelet packet decomposition on *x*_*j*−1,*m*_(1 ≤ *j* ≤ *i*);
3. The *x_j,n_* acquired by decomposition was solved for its normalized *l^p^*;
4. The predicting operators and updating operators corresponding to the minimal ||*x*_*j*−1,*m*_||*_p_* were selected as the optimal operators of *x*_*j*−1,*m*_;
5. The above steps (2)–(4) were repeated till layer *i* was decomposed completely.

### 2.4. Problems of Frequency Alias and Band Interlacing

There were two important problems in the lifting wavelet transform: frequency alias and band interlacing. The results of signal processing may be affected somewhat if such problems were ignored. Therefore, they were analyzed one by one and solved in this study.

#### 2.4.1. Frequency Alias

The same as the classic wavelet transform, the frequency alias also existed in the lifting wavelet transform. There were two causes for this [18]:

1. The subdivision step in the lifting algorithm was a down-sampling course, and the sampling rate of detail signals in the wavelet packet decomposition would no longer satisfy the Nyquist sampling principle. Therefore, the frequency alias occurred with $\frac{f_{s}}{2^{j+1}}$ (*f_s_* was the sampling frequency, and *j* was the number of decomposed layers) as the center of symmetry and the false frequency components was produced;
2. The undesirable cut-off characteristics of the high-pass filter and low-pass filter corresponding to predicting operators and updating operators made the frequency components of other nodes within the transitional zone of the filter to be folded up with the frequency boundary $\left[\frac{v}{2^{j+1}}\;\;f_{s},\;\frac{v+1}{2^{j+1}}\;\;f_{s}\right]$ (*f_s_* was the sampling frequency, *j* was the number of decomposed layers, and *v* = 0,1,⋯2*^j^* − 1) of the node as the center of symmetry.

For the frequency alias induced by cause (1), there were two solutions:

1. Single-branch reconstruction was performed on node signals, so that the folded frequencies in the decomposition could be folded back during the reconstruction;
2. The split step in the decomposition course and the induced down-sampling problem were removed, with the redundant lifting wavelet transform brought in.

The solution to the problem of frequency alias induced by cause (2) was as follows:

1. With the redundant lifting wavelet packet transform being performed on signal *x*, all node signals *x_j, k_* (*n*) (*j* indicated the number of the layer being decomposed currently; *k* = 1,2,⋯2*^j^* represented the serial numbers of nodes; *n* = 1,2,⋯*L*, *n* indicated the serial number of sampling point of *x_j,k_* (*n*) and *L* indicated the sample length of *x_j,k_* (*n*)) were acquired;
2. *FFT* transformation was performed on all *x_j,k_* (*n*):
  (10) $$X_{j,k}\;(m)=\sum_{n=0}^{L-1}x_{j,k}\;(n)\;W_{L}^{\mathrm{mn}}\;\;\;\;m=0,1,\cdots L-1$$
  where $W_{L}=e^{-j\frac{2\pi }{L}}$.
3. The frequency components excluding the band where *x_j,k_* (*n*) was set to zero:
  (11) $$\left\{ \begin{array}{ll} {\tilde{X}}_{j,k}\;(m)=X_{j,k}\;(m), & X_{j,k}\;(m)\in [\frac{k-1}{2^{j+1}}\;f_{s},\;\frac{k}{2^{j+1}}\;f_{s}]\\ {\tilde{X}}_{j,k}\;(m)=0, & \mathrm{others} \end{array}\right.$$
4. *IFFT* transformation was performed on *X̃_j,k_* (*m*) which was obtained through related processing:
  (12) $${\tilde{x}}_{j,k}\;(n)=\frac{1}{L}\sum_{n=0}^{L-1}{\tilde{X}}_{j,k}\;(m)\;W_{L}^{-\mathrm{mn}}\;\;\;m=0,\;1,\;\cdots L-1$$

In this study, the problem of frequency alias in the lifting algorithm was solved with the above method.

#### 2.4.2. Band Interlacing

There was the band interlacing in the lifting algorithm apart from the problem of frequency alias. Although the frequency alias induced by the course of subdivision down-sampling could be overcome with the redundant lifting algorithm, the problem of band interlacing still could not be solved. A simulation signal was given as follows: the redundant lifting wavelet packet decomposition was performed on the above simulation signal, and the result was as follows:

(13) s=sin(2*π*120*t)+sin(2*π*160*t)+sin(2*π*360*t)+sin(2*π*400*t)

**Figure 2.**
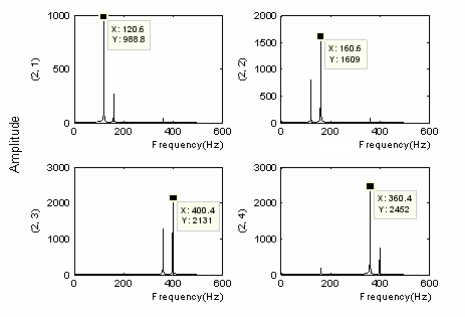
Node spectrogram about redundant lifting wavelet packet decomposition of simulation signal.

It was seen that the frequency interchange still occurred at nodes (2,3) and (2,4) in Figure 2. Accordingly, the partially induced down-sampling as well as the consequent frequency folding-up against the center of symmetry were not the primary causes of the band interlacing; instead, the primary reason was the frequency alias caused by the undesirable cut-off characteristics of filters. With the multi-layer redundant lifting wavelet packet decomposition being performed on signals (taking triple-layer decomposition for instance), the sequence of the nodes in different layers could be obtained as follows:

**Figure 3.**
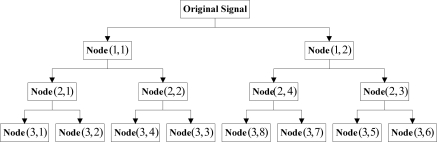
Node sequence in redundant lifting wavelet packet decomposition.

**Figure 4.**
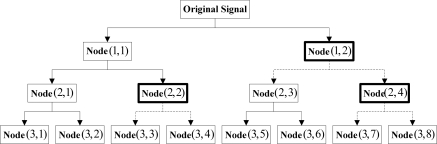
Schematic solution to band interlacing.

It is clear from Figure 3 that the decomposition results for all layers exhibited band interlacing from layer 2 on. Besides, it was also noticed from the figure that the occurrence of the interlacing had certain regularity, *i.e.*, the two nodes obtained would be interchanged when the high-frequency nodes at each layer were decomposed. Accordingly, the problem of band interlacing could be solved with the following method: when the wavelet packet decomposition was performed on the high-frequency nodes in each layer, the information of the two obtained nodes (high-frequency and low-frequency) was exchanged. As the above process proceeded successively in each layer, the decomposition results with theoretical sequential arrangement of nodes could be acquired eventually. Figure 4 below showed the schematic solution to band interlacing:

According to Figure 4, the original signals were decomposed at the first layer and required no interchanging; when it came to the second layer, decomposition results (2,4) and (2,3) were interchanged to (2,3) and (2,4) because (1,2) was a high-frequency signal, and a sequential result of (2,1), (2,2), (2,3) and (2,4) was obtained finally; when the original signals were decomposed at the third layer, decomposition results (3,4) and (3,3) were interchanged to (3,3) and (3,4), and (3,8) and (3,7) were interchanged to (3,7) and (3,8) because both nodes (2,2) and (2,4) were high-frequency signals; finally, a sequential decomposition result was obtained. The method above was applied layer by layer till the decomposition was finished. According to the discussions in the above sections, the improved forward transform of nonlinear redundant lifting wavelet packets proposed in this study was implemented as shown in the following figure:

**Figure 5.**
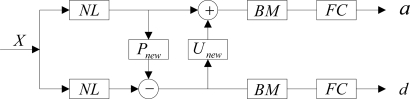
Block diagram of improved forward transform of nonlinear redundant lifting wavelet packets.

where *NL* was a nonlinear operator based on norm *l^p^* (*p* ≤ 1) and was used to select the optimal predicting operator and updating operator adaptively which matched to the characteristics of node signals; *P_new_* and *U_new_* were the redundant predicting operator and the updating operator, respectively; *BM* was the operator to solve the band interlacing; *FC* was the operator used to eliminate the frequency alias. After approximation signal *a* and detail signal *d* were acquired through the implementation of the above forward transform on signal *X* which was to be decomposed, the decomposition of nonlinear redundant lifting wavelet packets could be achieved with the above forward transform being repeated on *a* and *d*.

### 2.5. Node-Signal Single-Branch Reconstruction Algorithm

In order to extract characteristic information from the interested bands, the nonlinear redundant lifting wavelet packet single-branch reconstruction algorithm was applied to node signals. The specific implementation of the reconstruction was as follows:

1. The node information to be reconstructed was preserved, while all other node information was set to zero;
2. Since other node information was set to zero, the frequency alias induced by the undesirable cut-off characteristics of the filter could be ignored;
3. In the redundant lifting wavelet packet decomposition, the information about two nodes obtained from high-frequency signal decomposition was interchanged to solve the problem of band interlacing. Therefore, this course must be taken into account in reconstruction; otherwise, wrong reconstruction results would be obtained. In this study, an approach of recording decomposition paths was employed to record the decomposition paths of nodes in the wavelet packet decomposition, and reverse reconstruction was carried out based on the decomposition paths of nodes in single-branch reconstruction;
4. The predicting operators and updating operators chosen for the decomposition of nodes were also recorded, and reverse reconstruction was carried out based on the recording results in single-branch reconstruction because a nonlinear algorithm was used in decomposition.

**Figure 6.**
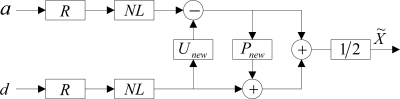
Block diagram about node-signal single-branch reconstruction of improved nonlinear redundant lifting wavelet packets.

The implementation flow of the above node-signal single-branch reconstruction algorithm was as shown in Figure 6. In the figure, *R* is the operator to record the decomposition paths of nodes; *NL* is a nonlinear operator. The node information was preserved while the information of all other nodes was set to zero in the single-branch reconstruction of a node (e.g., in the reconstruction of *a*, *d* was set to zero; *vice versa*).

Nodes were reconstructed according to recorded decomposition path *R* and nonlinear operator *NL*, and then the two outputs were averaged, with the result as the eventual output of the node in the reconstruction of the layer. With the above course repeated, the final result of the node-signal single-branch reconstruction in multi-layer decomposition could be obtained.

## 3. Characteristic Extraction Algorithm

In order to successfully extract the faint fault characteristics from strong background noise and achieve an effective fault diagnosis of mechanical equipment, the nodes obtained from the nonlinear redundant lifting wavelet packet decomposition must be processed further combined with the fault mechanisms of corresponding parts.

A spectral peak group with concentrated energy will be formed in a certain high-frequency band because of the modulating characteristics of the fault signals of roller bearings induced by resonance. Generally, great attention is paid to the band where the spectral peak group lies, due to the abundant fault information contained in it. Through the redundant lifting wavelet packet transform, signals are decomposed into different bands. In order to identify the node whose band is involved the spectral peak group, the analysis of the wavelet packet energy should be conducted according its energy concentration characteristics. Suppose *x_j,n,k_* is element No. *k* (*k* = 1,2,⋯*l*,) in node *n* (*n* = 1,2,⋯2*^j^*) at layer *j* and *l* is the sample length at the node, then the energy of the normalized wavelet packet is defined as follows:

(14) $$E(x_{j,n,k})=\sum_{k=1}^{l}x_{j,n,k}^{2}/\sum_{n=1}^{2^{j}}\sum_{k=1}^{l}x_{j,n,k}^{2}$$

A node with the maximal *E*(*x_j,n,k_*) was selected for single-branch reconstruction according to the algorithm in Section 2.5. Hilbert modulation and envelope spectrum analysis [19] were conducted on *x̃*(*t*) so as to identify the characteristic frequency of the fault because signal *x̃*(*t*) still contained high-frequency modulating information after single-branch reconstruction:

(15) $$\left\{ \begin{array}{l} H[\tilde{x}(t)]=\frac{1}{\pi }\int_{-\infty }^{\infty }\frac{\tilde{x}(\tau )}{t-\tau }d\tau \\ \tilde{X}(t)=\tilde{x}(t)+\mathrm{iH}[\tilde{x}(t)]\\ A[\tilde{X}(t)]=\left|\tilde{X}(t)\right|=\sqrt{{\tilde{x}}^{2}(t)+H^{2}[\tilde{x}(t)]} \end{array}\right.$$

In the equation, *H*[*x̃*(*t*)], *X̃*(*t*) and *A*[*X̃*(*t*)] are the Hilbert transform, analytic form and amplitude envelop of *x̃*(*t*), respectively. In conclusion, the fault characteristics extraction algorithm used in this study proceeded as follows:

1. The improved nonlinear redundant lifting wavelet packet transform was performed on signals;
2. The wavelet packet energy analysis was performed on the nodes obtained from decomposition;
3. Single-branch reconstruction, Hilbert modulation and envelop spectrum analysis were conducted on the nodes corresponding to the maximal energy.

## 4. Engineering Cases Analysis

The improved algorithm for nonlinear redundant lifting wavelet packet was applied to the case analysis on the step-up boxes in the high-speed finishing mills of a steel mill. The driving chain of the finishing mill is shown in the following figure:

**Figure 7.**
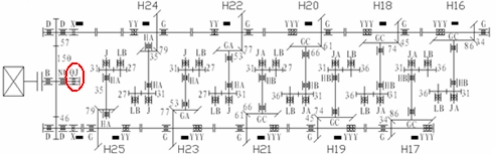
Driving chain of finishing mill in a steel mill.

In the figure, black stripes represented the locations of measurement points. The on-line monitoring system detected that the peaks at the horizontal measurement point at the southern output terminal of the step-up box (marked by a red ellipse in Figure 7) exhibited an increasing trend from March 3, 2009, with the maximum of 140.121 m/s^2^. The vibration acceleration signals (with the sampling frequency of 10,000 Hz and the number of sampling points of 2,048) at the measurement point at 3:00, February 22 were selected for time-domain and frequency-domain analysis. The results were as follows:

**Figure 8.**
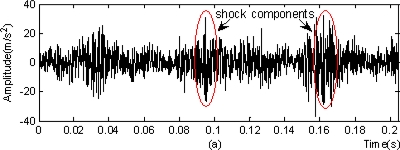

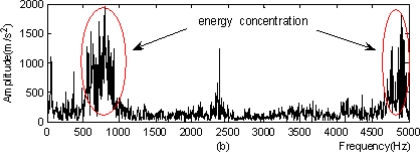
Basic analysis of vibration acceleration signals at measurement point: **(a)** time-domain image. **(b)** frequency spectrogram.

From the time-domain image, it was clear that there were shock components and energy concentration also appeared in the frequency spectrogram. Accordingly, it was deduced preliminarily that the step-up box may have the potential for failure.

The method in this study was applied to the signal for triple-layer wavelet packet decomposition, with the result obtained as follows: the calculated values of norm *l^p^* for all nodes in all layers are shown in the following Table:

**Table 2.** *l^p^* (×10^29^) of all nodes.

**Nodes**	(0,1)	(1,1)	(1,2)	(2,1)	(2,2)	(2,3)	(2,4)
**Operators**							
(4,4)	8.4610	10.029	9.7141	10.466	10.430	10.288	9.9694
(12,4)	8.4799	9.9424	9.7322	10.443	10.419	10.300	9.9515
(12,12)	8.4499	9.9080	9.7571	10.438	10.425	10.300	9.9772
(20,4)	8.4866	9.9333	9.7375	10.438	10.413	10.314	9.9509
(20,12)	8.4482	9.9071	9.7595	10.434	10.421	10.312	9.9774
(20,20)	8.4536	9.9088	9.7654	10.423	10.425	10.308	9.9871

According to Table 2, the optimal predicting operator and updating operator used for the further wavelet packet decomposition of nodes were as follows:

**Table 3.** Optimal predicting operator and updating operator for nodes.

**Nodes**	(0,1)	(1,1)	(1,2)	(2,1)	(2,2)	(2,3)	(2,4)
**Operators**	(20,12)	(20,12)	(4,4)	(20,20)	(20,4)	(4,4)	(20,4)

**Figure 9.**
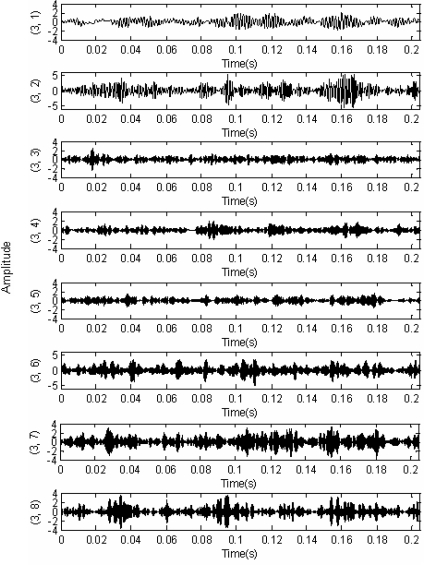
Triple-layer nonlinear redundant lifting wavelet packet decomposition of signals.

**Figure 10.**
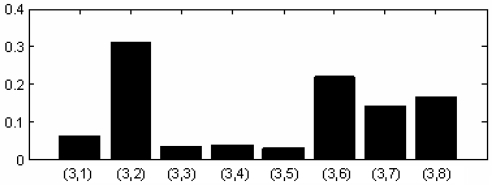
Wavelet packet energy analysis.

According to Table 3, the optimal predicting operator and updating operator were applied for the nonlinear redundant lifting wavelet packet decomposition and thus the time-domain images for eight nodes obtained by triple-layer wavelet packet decomposition were shown as indicated in Figure 9. The normalized wavelet packet energy was taken from the eight nodes in Figure 9, and the results are shown in Figure 10.

From the wavelet packet energy shown in Figure 10, distribution and comparison of the energy of the eight nodes could be seen. The energy corresponding to node (3,2) was maximal, so the single-branch reconstruction and Hilbert modulation were carried out on (3,2). In order to verify the superiority of the method in this study, the local frequency spectrograms of signals were selected with both results being compared, as follows:

**Figure 11.**
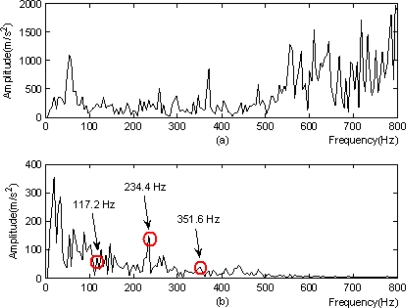
Modulation analysis: **(a)** local frequency spectrogram of signals. **(b)** modulation spectrogram after single-branch reconstruction of nodes.

From the analysis of the results in Figure 11, several conclusions were made:

1. Figure 11(b) suggested the frequency component of *117.2 Hz* as well as its double frequency 234.4*Hz* and triple frequency *351.6 Hz*, and the component of double frequency was distinct;
2. The above frequencies could not be found in Figure 11(a);
3. The method in this study was superior according to the above comparison;
4. The base frequency of *117.2 Hz* in the figure was very close to the calculated characteristic frequency *119.5 Hz* of the fault occurred on the outer ring of a horizontal bearing at the southern output terminal of the step-up box of the finishing mill, within the range of frequency resolution.

**Figure 12.**
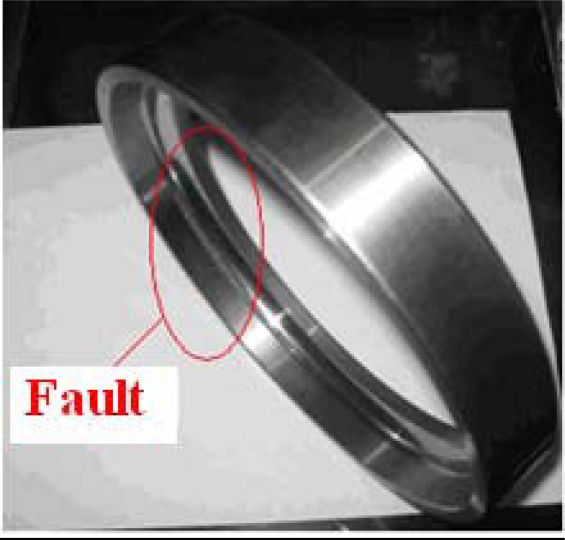
Schematic damage of bearing outer-ring of axis I at the southern output terminal of the step-up box.

It was deduced that the bearing had a fault on its outer ring. This analysis result agreed completely with the result of unboxed overhaul in mid-March of 2009. The image in Figure 12 shows the bearing damage detected in the overhaul.

## 5. Conclusions

In this study, an improved algorithm for nonlinear redundant lifting wavelet packets was put forward and applied to the extraction of faint fault characteristics. With the minimal norm *l^p^* as the criterion for selecting the optimal predicting operator and updating operator which matched the characteristics of node signals, the redundant lifting wavelet packet decomposition were performed on different nodes through predicting operators and updating operators with different vanishing moments. The frequency alias and band interlacing emerged during decomposition were analyzed, and a solution was given. The node signals were selected for single-branch reconstruction and Hilbert modulation based on the wavelet packet energy method. With the application of the method described in this study to the case of outer-ring damage in the bearing of a step-up box of a finishing mill from a steel mill, the characteristic frequency and the frequency multiplication component of the outer-ring faults of bearings were extracted successively in the analysis results, proving the feasibility and validity of the method in the fault diagnosis of roller bearings.

## References

[1] Sweldens W. (1996). The lifting scheme: A custom-design construction of biorthogonal wavelet. Appl. Comput. Harmonic. Anal.

[2] Sweldens W. (1997). The lifting scheme: A construction of second generation wavelet constructions. SIAM J. Math. Aanal.

[3] Claypoole R.L., Baraniuk R.G., Nowak R.D. Adaptive wavelet transforms via lifting.

[4] Duan C.D., He Z.J., Jiang H.K. (2007). A sliding window feature extraction method for rotating machinery based on the lifting scheme. J. Sound. Vib.

[5] Huang Y.X., Liu C.L., Zha X.F., Li Y.M. (2009). An enhanced feature extraction model using lifting-based wavelet packet transform scheme and sampling-importance-resampling analysis. Mech. Syst. Signal. Process.

[6] Samuel P.D., Pines D.J. (2009). Constrained adaptive lifting and the CAL4 metric for helicopter transmission diagnostics. J. Sound. Vib.

[7] Lee C.S., Lee C.K., Yoo K.Y. (2000). New lifting based structure for undecimated wavelet transform. Electron. Lett.

[8] Li Z., He Z.J., Zi Y.Y., Chen X.F. (2008). B*earing condit*ion moni*tor*ing based on shock pulse method and improved redundant lifting scheme. Math. Comput. Simulat.

[9] Zhou R., Bao W., Li N., Huang X., Yu D.R. (2010). Mechanical equipment fault diagnosis based on redundant second generation wavelet packet transform. Digit. Signal. Process.

[10] Daubechies I., Sweldens W. (1998). Factoring wavelet transforms into lifting steps. J. Fourier. Anal. Appl.

[11] Claypoole R.L., Baraniuk R.G. Flexible Wavelet Transforms Using Lifting. http://scholarship.rice.edu/handle/1911/19805.

[12] Yang F.S. (1999). Analysis and Application Using Wavelet Transform.

[13] Duan C.D. (2004). Research on Fault Diagnosis Techniques Using Second Generation Wavelet Transform.

[14] Claypoole R.L., Geoffrey D., Sweldens W., Baraniuk R.G. (2003). Nonlinear wavelet transforms for image coding via lifting. IEEE Trans. Image. Process.

[15] He Z.J., Zi Y.Y., Zhang X.N. (2007). Present Signal Processing and Engineering Application.

[16] Cheng F.B., Tang B.P., Zhong Y.M. (2008). Filter denoising method based on optimal Morlet wavelets and SVD and its application to fault diagnosis. Shock. Vib.

[17] Karvanen J., Cichocki A. Measuring sparseness of noisy signals.

[18] Bao W., Zhou R., Yang J.G., Yu D.R., Li N. (2009). Anti-aliasing lifting scheme for mechanical vibration fault feature extraction. Mech. Syst. Signal. Process.

[19] Ding K., Li W.H., Zhu X.Y. (2006). Practical Technology for Gear and Gearbox Fault Diagnosis.

